# Molecular Characterization of a Novel Positive-Sense, Single-Stranded RNA Mycovirus Infecting the Plant Pathogenic Fungus *Sclerotinia sclerotiorum*

**DOI:** 10.3390/v7052470

**Published:** 2015-05-21

**Authors:** Rong Liu, Jiasen Cheng, Yanping Fu, Daohong Jiang, Jiatao Xie

**Affiliations:** State Key Laboratory of Agricultural Microbiology, The Provincial Key Lab of Plant Pathology of Hubei Province, College of Plant Science and Technology, Huazhong Agricultural University, Wuhan 430070, China; E-Mails: rongliu@webmail.hzau.edu.cn (R.L.); jiasencheng@mail.hzau.edu.cn (J.C.); yanpingfu@mail.hzau.edu.cn (Y.F.); daohongjiang@mail.hzau.edu.cn (D.J.)

**Keywords:** *Sclerotinia sclerotiorum*, mycovirus, fusarivirus, *Fusarium graminearum* virus 1, *Rosellinia necatrix* fusarivirus 1

## Abstract

Recent studies have demonstrated that a diverse array of mycoviruses infect the plant pathogenic fungus *Sclerotinia sclerotiorum*. Here, we report the molecular characterization of a newly identified mycovirus, Sclerotinia sclerotiorum fusarivirus 1 (SsFV1), which was isolated from a sclerotia-defective strain JMTJ14 of *S. sclerotiorum*. Excluding a poly (A) tail, the genome of SsFV1 comprises 7754 nucleotides (nts) in length with 83 and 418 nts for 5'- and 3'-untranslated regions, respectively. SsFV1 has four non-overlapping open reading frames (ORFs): ORF1 encodes a 191 kDa polyprotein (1664 amino acid residues in length) containing conserved RNA-dependent RNA polymerase (RdRp) and helicase domains; the other three ORFs encode three putative hypothetical proteins of unknown function. Phylogenetic analysis, based on RdRp and Helicase domains, indicated that SsFV1 is phylogenetically related to *Rosellinia necatrix* fusarivirus 1 (RnFV1), Fusarium graminearum virus-DK21 (FgV1), and Penicillium roqueforti RNA mycovirus 1 (PrRV1), a cluster of an independent group belonging to a newly proposed family Fusarividae. However, SsFV1 is markedly different from FgV1 and RnFV1 in genome organization and nucleotide sequence. SsFV1 was transmitted successfully to two vegetatively incompatible virus-free strains. SsFV1 is not responsible for the abnormal phenotype of strain JMTJ14.

## 1. Introduction

Based on their genomic nature, all reported fungal viruses (or mycoviruses) have been divided into four groups: double-stranded RNA (dsRNA), positive-sense single-stranded RNA (+ssRNA), negative-sense single-stranded RNA (-ssRNA), and single-stranded DNA (ssDNA) [1,2]. Mycoviruses with double stranded DNA have not been discovered in fungi. +ssRNA mycoviruses are now classified into seven families *Alphaflexiviridae*, *Barnaviride*, *Endornaviridae*, *Gammaflexiviridae*, *Hypoviridae*, *Narnaviridae*, and newly proposed Fusariviridae [3,4,5]. With the exception of two mycoviruses, Botrytis virus F and Botrytis virus X [6], the other previously reported +ssRNA mycoviruses in phytopathogenic fungi do not possess coat protein genes and lack true virions in their life cycles. Although an increasing number of +ssRNA mycoviruses were characterized at the genome level, most of them have not been assigned to formal taxa [3,4,7]. Screening of more +ssRNA mycoviruses contributes to a better understanding of their roles in virus evolution or help in establishing new families for accommodating those unsigned mycoviruses. Moreover, fully elucidating the ecology and evolution of these mycoviruses and new types of mycoviruses is critical to progress in mycovirology.

The ascomycete fungus *Sclerotinia sclerotiorum* is an important ubiquitous necrotrophic pathogen that attacks over 400 plant species and is responsible for reducing the annual yield of a wide range of economically important crops [8]. This pathogen produces sclerotia that play a major role in its life and infection cycles. Since the dsRNA factor was first reported in hypovirulent strain 91 of *S. sclerotiorum*, mycoviruses possessing diverse genomes of mostly ssRNA and dsRNA and recently circular ssDNA, have been described and most of those reported mycoviruses cause hypovirulence [1,2,9,10]. The first reported ssDNA mycovirus, Sclerotinia sclerotiorum hypovirulence-associated DNA virus 1 (SsHADV-1), was isolated from the hypovirulent strain DT-8 and is being exploited as a potentially effective biocontrol agent for Sclerotinia diseases [11,12]. The first reported -ssRNA mycovirus, Sclerotinia sclerotiorum negative sense RNA virus 1 (SsNsRV-1), was isolated from a hypovirulent strain AH98 and is related to members of *Nyamiviridae* and *Bornaviridae* [2]. Two partitiviruses, Sclerotinia sclerotiorum partitivirus S (SsPV-S) and SsPV1, were characterized. Phylogenetic analysis of coat protein of the SsPV-S, isolated from the virulent strain Sunf-M, reveals that horizontal gene transfer events are widespread and range from dsRNA viruses to eukaryotic nuclear genomes [13,14]. SsPV1 does not only confer hypovirulence on both *S. sclerotiorum* and *Botrytis cinerea* strains, but also has strong infectivity to break the obstacle of vegetative incompatibility [15]. +ssRNA mycoviruses are the most common in population of *S. sclerotiorum* and at least ten +ssRNA mycoviruses have been described in this phytophathogenic fungus. *S. sclerotiorum* strain Ep-1PN harbors two +ssRNA mycoviruses, Sclerotinia sclerotiorum debilitation-associated RNA virus (SsDRV) and Sclerotinia sclerotiorum RNA virus L (SsRV-L) [6,16]. SsDRV is the first well-characterized mycovirus that is associated with hypovirulence of *S. sclerotiorum* [16], whereas SsRV-L is related to the human pathogen hepatitis E and *rubi-like* viruses [7]. Seven mitovirus species (twelve isolates) have, thus far, been reported in *S. sclerotiorum*. Two mitoviruses (Sclerotinia sclerotiorum mitoviruses 1 and 2, SsMV1 and SsMV2) were isolated from a US hypovirulent strain KL-1 and from a Chinese hypovirulent strain [10,17], and a mixture of six mitoviruses (SsMV2 to SsMV7) were identified in four New Zealand strains [18,19]. The hypovirus Sclerotinia sclerotiorum hypovirus 1 (SsHV1) and its satellite-like RNA co-infected the hypovirulent strain SZ-150, and the satellite-like RNA has a key role in conferring hypovirulence to *S. sclerotiorum* [20]. SsHV2 has been identified in three Sclerotinia strains collected from difference continents and its genomic features are greatly different from previously reported hypoviruses [21,22,23]. Sclerotinia sclerotiorum endornavirus 1 (SsEV1) was characterized and was associated with latent infection of *S. sclerotiorum* [24]. Therefore, these mycovirus-rich resources will enhance opportunities to understand viral ecology, evolution and the enrichment of virocontrol biological resources.

In the present study, we describe a novel +ssRNA mycovirus, Sclerotinia sclerotiorum fusarivirus 1 (SsFV1), isolated from strain JMTJ14. Three objectives were intended for this research. Firstly, to isolate and characterize the genome of SsFV1. Secondly, to elucidate the phylogenetic relationships between SsFV1 and other reported viruses based on RNA-dependent RNA polymerase (RdRp) sequences. Thirdly, to investigate the impact of SsFV1 on the biology of *S. sclerotiorum*.

## 2. Materials and Methods

### 2.1. Fungal Strains and Culturing

Strain JMTJ14 was isolated from a sclerotium collected from a diseased rapeseed in Jingmen county, Hubei province, People's Republic of China. Strain JMTJ14 does not produce any sclerotia on Potato Dextrose Agar (PDA) or carrot medium. Amplification and sequencing of rDNA gene fragment of ITS (internal transcript spacer), however, confirmed that strain JMTJ14 is a sclerotia-defective strain of *S. sclerotiorum*. Two virus-free highly virulent strains, EP-1PNA367 and 1980, were also used in this study. EP-1PNA367R and 1980R were labeled with a hygromycin-resistance gene using an *Agrobactirium tumfacience*-mediated transformation method. Mycelial compatibility between strains of *S. sclerotiorum* was tested on PDA containing McCormick’s red food coloring with a concentration of 75 μL/L [25]. A visible interaction zone shows a line of fluffy or inhibited zone between vegetatively incompatible groups, such as strain JMJT14 *vs.* Ep-1PNA367R or 1980R, whereas an interaction zone does not appear between vegetatively compatible groups, such as Ep-1PNA367R *vs.* Ep-1PNA367R (unpublished data). All *S. sclerotiorum* strains were grown on PDA at 20–22 °C and stored on PDA slants at 4–6 °C.

### 2.2. dsRNA Purification

For dsRNA extraction, strain JMTJ14 was cultured on PDA plates covered with cellophane membranes for 3 days. The mycelium was collected and ground to a fine powder in liquid nitrogen with a mortar with a pestle, and dsRNA was isolated with CF-11 cellulose (Sigma-Aldrich, Dorset, UK) in the presence of 16%–18% ethanol as previously described [16]. The dsRNA preparation was treated with DNase I and S1 nuclease (TaKaRa, Dalian, China), and then the treated dsRNA was electrophorized and gel purified using a gel extraction kit (Axygene biosciences, Hangzhou, China) and stored at −80 °C.

### 2.3. Synthesis and Cloning of cDNA

The cDNA cloning strategy for dsRNA isolated from strain JMTJ14 was conducted as previously described by Xie *et al.* [20] using a cDNA synthesis kit (Fermentas, Ontario, Canada) with tagged random dN6 primers (5'-CGATCGATCATGATGCAATGCNNNNNN-3'). Approximately 500 ng of purified dsRNA was mixed with 1.2 μM random dN6 primers and 3 µL of dimethyl sulfoxide (DMSO), and diethyl pyrocarbonate (DEPC)-treated double-distilled H_2_O was added to a final volume of 12 μL. The mixture was heated to 95 °C for 10 min and chilled on ice for 5 min. First strand cDNA was synthesized according to manufacturer’s instructions for cDNA synthesis. After reverse transcription, random cDNA products were obtained using a single specific primer (5'-CGATCGATCATGATGCAATGC-3') based on the sequence of tagged random dN6 primers. Based on the cDNA sequence obtained with the random primer, six specific paired primers (Figure S1 and Table S1) were designed and a series of reverse transcription PCRs, which connect the initial random cDNA synthesis, were conducted to amplify portions of the dsRNA genome that was not cloned by the initial random cDNA synthesis (Figure S1). cDNA amplification was performed using a C100™ Thermal Cycler (Bio-Rad, Hercules, CA, USA) and the resultant PCR products were sequenced. Sequences of all cDNA clones were subjected to obtain via blast program analysis in NCBI website [26].

To obtain the complete viral genome sequence, 5'-RACE PCR was conducted as described by Potgieter *et al.* [27] to obtain 5' terminal sequence. The 3' terminus of each strand of dsRNA was ligated to the phosphorylated 5'-end oligonucleotide PC3-T7 loop (5'-p-GGATCCCGGGAATTCGGTAATACGACTCACTATATTTTTATAGTGAGTCGTATTA-OH-3') in presence of T4 RNA Ligase (TaKaRa) at 4–8 °C for 18 h. Oligonucleotide-ligated dsRNA was purified using AXYGEN gel extraction kit following manufacturer’s recommendations. The purified ligated dsRNA was then denatured in DMSO as described above and was used for a reverse transcription reaction in presence of reverse transcriptase. The reverse transcription reaction was incubated in a thermal cycler at 42 °C for 60 min followed by 45 °C for 15 min. The cDNA was amplified using a primer with a complementary sequence to the RNA ligation oligonucleotide PC2 (5'- CCGAATTCCCGGGATCC-3') and the 5' end special primer (5'-TGCTTTTCTCATAAACTTCATCCTC-3') or 3′ end special primer (5'-GTACAACCACATGATATCAGCA-3′) for terminal sequences of dsRNA, respectively. The expected PCR products from the cDNA sequence were fractionated by electrophoresis in an agarose gel and purified using a gel extraction kit. The PCR product was cloned into the pMD18-T Vector (TaKaRa) and sequenced. To ensure the accuracy of the obtained cDNA sequence, every nucleotide of the genomic dsRNA was determined by sequencing at least three independent overlapping clones.

### 2.4. Computer Analysis of Nucleic Acid and Deduced Protein Sequences

The sequences of selected viruses referenced in this paper (Table S1) were retrieved from the NCBI GenBank database [28] and used for comparative analyses. Motif searching was conducted using the Transfac [29] and InterProscan software [30]. Multiple sequence alignment was performed using the DNAMAN, CLUSTAL_W, MAFFT version 7 [31], and BLAST software [26]. On the basis of aligned sequences, phylogenetic trees were constructed using the Maximum Likelihood method with the MEGA version 6.0 program [32], and GTR (general time reversible) model with G + I (invariant sites and distributed range) was applied. Phylogenetic trees were plotted with TreeView and manually edited. 

### 2.5. Horizontal Transmission and Virus Elimination

To assay potential horizontal transmission of SsFV1 between strains of *S. sclerotiorum*, 0.05 g of fresh mycelial fragments from strain JMTJ14 was mixed with that from EP-1PNA367R or 1980R isolates on a PDA plate. After mixed-culturing for two weeks, mycelial plugs from the mixed mycelia of JMTJ14 and EP-1PNA367R or JMTJ14 and 1980R were transferred to a fresh PDA plate containing 30 μg/mL hygromycin. New colonies were sub-cultured five times on hygromycin PDA and then transferred onto fresh PDA without hygromycin. The biological properties of those subcultures were further assayed as previously described [33]. To assay the virulence of strain JMTJ14 and other isolates of *S. sclerotiorum*, actively growing mycelial plugs from individual strains were inoculated onto detached rapeseed leaves. Inoculated leaves were maintained in a 20 °C incubator for 3 days and then average lesion size was measured for analysis. Biological properties data were subjected to analysis of variance (ANOVA) using the SAS^®^ 8.0 program. The treatment means were compared using least significant difference (LSD) test at a *p* = 0.01 level.

To eliminate mycovirus from strain JMTJ14, several approaches were applied. These include protoplast isolation, hyphal tipping, chemotherapy, and combined two or three approaches. The procedures of protoplast isolation and hyhal tipping were conducted as previously described [33]. For chemotherapy, mycelial plugs of strain JMTJ14 were inoculated on PDA containing either ribavirin (0.5 mg/mL, 1 mg/mL or 2 mg/mL) or cycloheximide (355 µM) and cultured for one day. Hyphal tips were transferred to fresh PDA medium containing the same chemicals for three cycles of chemotherapy. All cultures from the different treatments were grown on PDA and generated mycelia were used for total RNA isolation.

### 2.6. Detection of SsFV1 in Individual Strains of S. sclerotiorum

Detection of SsFV1 in the different cultures was performed by dsRNA extraction, RT-PCR and Northern blot analysis. Northern hybridization analysis was performed as previously described with minor modifications [15]. Briefly, 100 ng dsRNA extracted from strain JMTJ14, EP-1PNA367RV, and 1980RV were separated on a 1% agarose gel with 80 V electrophoresis for 5 h. The gel was then soaked for 30 min in 0.1M NaOH and neutralized in 0.1 M Tris-HCl (pH 8.0) for 30 min. The RNA fragments in pre-treated gel were transferred to a pre-wetted nylon membrane (BIO-RAD) in 20 × SSC buffer for 16–24 h. and UV was used for cross-linking RNA to nylon membranes. Prehybridization and hybridization were carried out under high stringency conditions in a hybridization buffer (0.5 M NaCl, 4% blocking reagent). Post-hybridization washes were conducted twice with primary and secondary wash buffer as described in a CDP-Star kit (GE Healthcare, Life Sciences, UK). Hybridization signals were detected by chemiluminescence using a CDP-Star Detection kit (GE Healthcare, Life Sciences). All radioisotopic images were recorded using the Gel Doc XR+ imaging system with IMAGE GAUGE (BIO-RAD). The cDNA probe JRP680 corresponding to the SsFV1 sequences from nt positions 2307 to 2986 was obtained with specific primers (JRP680F: 5'-GGCACAGGATTAACAACTGGTCAT-3' and JRP680R: 5'-CCGTATTCAACAATGTCGTCAGGAT-3') and was labeled using an GEBlot Phototope kit (GE Healthcare, Life Sciences).

## 3. Results and Discussion

### 3.1. DsRNA Isolated from Strain JMTJ14 of S. sclerotiorum

**Figure 1:**
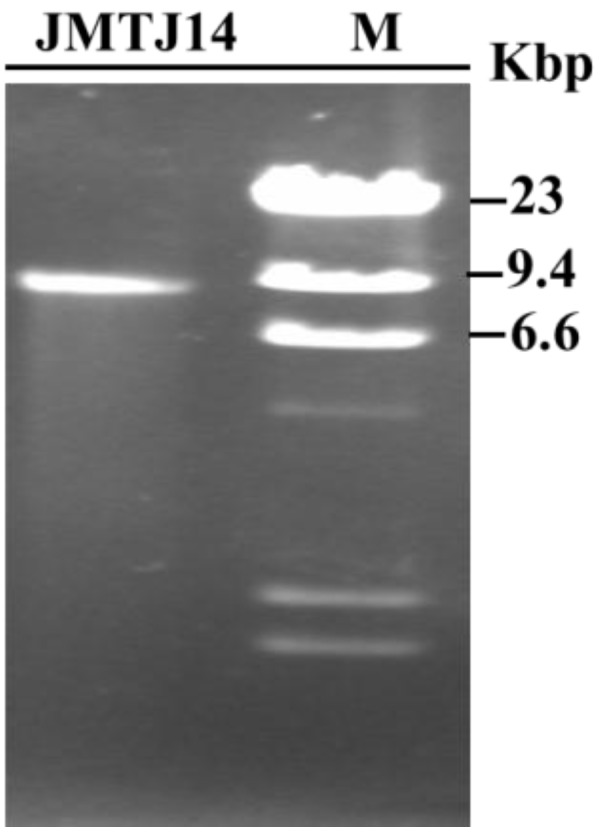
Agarose gel electrophoresis of dsRNA isolated from strain JMTJ14 of *S. sclerotiorum*. The dsRNA segment was treated with DNase I and S1 nuclease prior to fractionation on a 1.0% agarose gel (lane JMJT14). The size of the dsRNA was estimated using DNA size markers generated by digestion of λ DNA with *Hin*d III (lane M).

*S. sclerotiorum* strain JMTJ14 was originally isolated from a sclerotium collected from a diseased rapeseed. However, after hyphal tipping and subculturing, isolates from this strain lost the capability to produce sclerotia on PDA or carrot medium. To verify whether strain JMTJ14 is infected with mycoviruses, genomic DNA and dsRNA were extracted. The results suggested that no DNA of extra-chromosomal origin was evident in strain JMTJ14, but a dsRNA segment that was resistant to digestion with DNase I and S1 nuclease was detected from mycelia of strain JMTJ14 (Figure 1). This dsRNA segment was confirmed to be of viral origin in the present study and the virus tentatively named SsFV1.

### 3.2. Nucleotide Sequence and Genome Organization of SsFV1

**Figure 2:**
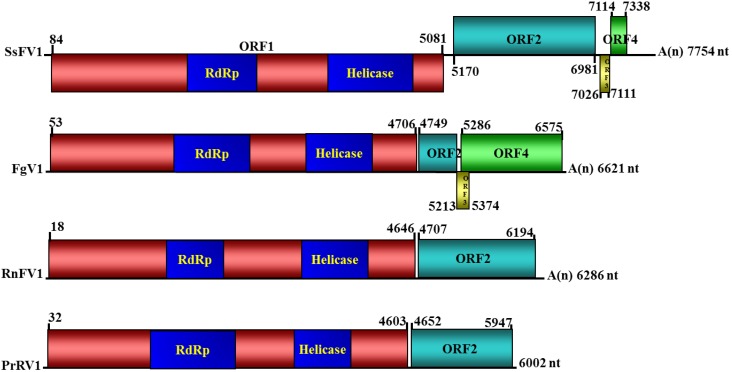
Diagrammatic representation of the putative genomic organization of SsFV1 and related mycoviruses Fusarium graminearum virus-DK21 (FgV1), Rosellinia necatrix fusarivirus 1 (RnFV1), and Penicillium roqueforti RNA mycovirus 1 (PrRV1). Open reading frames (ORFs) are highlighted by shading with different colors. ORFs drawn above and below solid lines refer to different frame for translation. The numbers above solid lines refer to map positions of initiation and termination codons of the respective ORFs. SsFV1 and FgV1 have four putative ORFs, RnFV1 and PrRV1 have two putative ORFs. ‘A(n)’ represents poly(A) structure. The relative positions of the RdRp (RNA-dependent RNA polymerase) and helicase domains in the polyprotein encoded by ORF1 are shaded in blue.

To obtain the full-length cDNA sequence of SsFV1 isolated from strain JMTJ14, more than twenty random cDNA clones were obtained and sequenced. Based on information from the obtained sequences, several specific primers were designed, and gap fragments were generated. The sequencing results revealed that these fragments aligned with each other based on their overlapping sequences. Three independent amplicons for the 5'- and 3'-terminal regions were obtained via classical RACE PCR procedures. The complete cDNA sequence was finally determined via sequence assembly using bioinformatics software. The full-length cDNA sequence of SsFV1 isolated from strain JMTJ14 was deposited in the GenBank database under the accession number KP842791. A schematic representation of the genomic organization of SsFV1 is shown in Figure 2.

The full-length cDNA of SsFV1 is 7754 nucleotides (nts) in length excluding the polyadenylate-poly(A)-tail. The base composition of the entire genome of SsFV1 is 32.3% A, 17.5% C, 21.2% G, and 29.0% U. These results reveal that SsFV1 has a rich A + U content (61.3%). The 5'- and 3'-untranslated regions (UTRs) of SsFV1 are 83- and 418-nts, respectively. The coding strand of SsFV1 contains four putative open reading frames (ORFs), of which two are large (ORF1 and ORF2) and two are small ORFs (ORF3 and ORF4). No large ORFs were found in the opposite orientation. 

ORF1 possesses an AUG codon at nt 84 and extends to UAG codon at nt 5081 (Figure 2). ORF1 encodes a 1664 amino acid (aa) polyprotein with an approximate molecular mass of 191 kDa. Comparison to known-viral sequences using the conserved domain search of the NCBI database showed that the complete protein sequence predicted to be encoded by ORF1 of SsFV1 contains two conserved sequence domains: an RdRp ( aa position from 584 to 857) and a helicase (aa positions from 1178 to 1536). ORF2 is in a different frame from ORF1. ORF2 possesses an AUG codon at nt 5169 and extends to UAG at nt 6981. ORF2 encodes a 602 aa protein with an approximate molecular mass of 68 kDa. ORFs 3 and 4, which are proximal to the 3'-terminal region, encode two putative smaller proteins of 27 aa and 73 aa, respectively. No conserved domains were detected in motif library based on ORF2, ORF3, or ORF4-encoded protein sequences.

### 3.3. Sequence Comparison and Phylogenetic Analysis of Conserved Domains from the SsFV1

A Blastp search of the NCBI protein database was performed using the full-length amino acid sequence of ORF1-encoded protein of SsFV1. The result showed that SsFV1 has a high similarity to three mycoviruses: Penicillium roqueforti SsRNA mycovirus 1 (PrRV1, 34% identity), Fusarium graminearum virus-DK21 (FgV1, 35% identity), and Rosellinia necatrix fusarivirus 1 (RnFV1, 33% identity). Motif scan for ORF1-encoded protein reveals that this putative protein contains a typical RdRp region and an RNA helicase domain. To more extensively determine the degree of relatedness between SsFV1 and other known viruses, alignment and phylogenetic analysis of two conserved domains were performed. 

**Figure 3:**
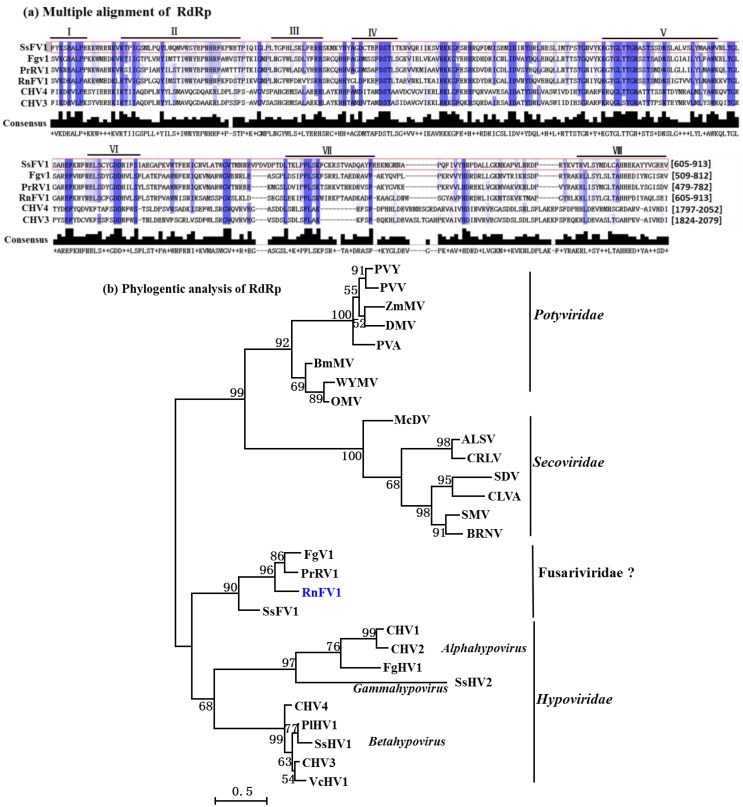

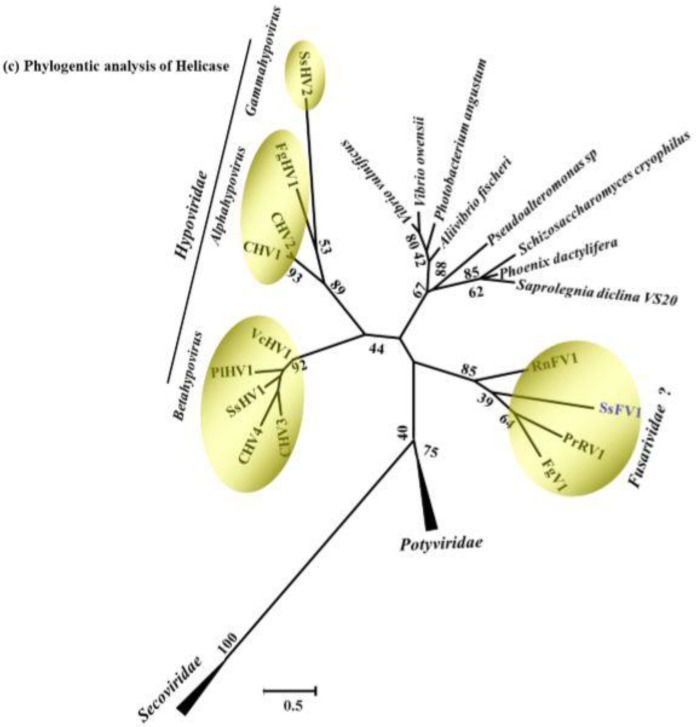
Multiple alignment and phylogenetic analysis of the conserved domains in SsFV1 and several selected viruses. (**a**) Multiple alignment of the aa sequences of the putative RdRp domains of SsFV1, FgV1, RnFV1, PrRV1, CHV3, and CHV4. The default color scheme for percentage identity in the Jalview program was used. Consensus is the consensus level for the multiple alignments. Number (I-VIII) refers to the eight conserved motifs characteristic of RdRps of RNA viruses. The number in square brackets refers region of RdRp domain in polyprotein encoded by the referenced mycoviruses; (**b**) an ML phylogenetic tree constructed based on alignment of the aa sequences of the conserved motifs in the RdRp domain in SsFV1 and selected viruses from families *Hypoviridae*, *Potyviridae*, and *Secoviridae*; (**c**) phylogenetic analysis of SsFV1, selected RNA viruses, and helicase-like genes of fungi and bacterium, based on an ML tree inferred from the helicase domain. Bootstrap values (%) obtained with 1000 replicates are indicated on the branches, and branch lengths correspond to genetic distance; the scale bar at lower left corresponds to a genetic distance of 0.5 for RdRp and helicase domain analysis. SsFV1 reported in the present phylogenetic tree was emphasized with blue. All abbreviations for virus names, viral protein accession numbers, and sequence identities are shown in Table S2.

RdRp domain of SsFV1 was aligned with that of five selected viruses [PrRV1, FgV1, RnFV1, Cryphonectria hypovirus 3 (CHV3), and Cryphonectria hypovirus 4 (CHV4)] using the ClustalX (Ver.1.81) program (Figure 3a). Multiple alignments revealed that eight conserved motifs were detected in RdRp domain of SsFV1 that share 48-52% aa sequence identities with corresponding regions of PrRV1, FgV1, and RnFV1. Similar to previously reported results for FgV1 and RnFV1, the RdRp region of SsFV1 also has a significant sequence similarity (25% to 35% aa sequence identities) to the RdRps encoded by members of families *Hypoviridae*, *Potyviridae*, and *Secoviridae* (Table S1). The phylogenetic tree of the RdRp domain further supported the conclusion that the cluster of SsFV1, PrRV1, FgV1, and RnFV1 formed an independent branch that was distant from members of *Hypoviridae* and +ssRNA plant viruses (Figure 3b).

The conserved helicase domain (aa positions 1178 to 1536) is located in the C-terminal region of the putative ORF1-encoded polyprotein of SsFV1. This helicase domain shares high sequence identity with that of FgV1 (33%), RnFV1 (34%) and PrRV1 (34%), but has little or no sequence identities with members of *Hypoviridae*. It is notable that the helicase domain of SsFV1 also has sequence similarity (21% to 26% identities) to helicase-like sequences from fungi and bacteria (Table S2). A phylogenetic tree was constructed based on multiple alignment of the SsFV1 helicase domain and its homologous sequences (Figure 3c). As expected, SsFV1 formed an independent branch with PrRV1, FgV1, and RnFV1 and this phylogenetic branch was more closely related to members of *Hypoviridae*, which was consistent with the RdRp domain alignment analysis.

Thus, taking together the results of genomic organization and phylogenetic tree, SsFV1 is most closely related to three viruses (host fungus in parenthesis): FgV1 (*Fusarium graminearum*), RnFV1 (*Rosellinia necatrix*), and PrRV1 (*Penicillium roqueforti*). These four viruses clustered together in an independent phylogenetic branch, which is closely related to members of *Hypoviridae*, *Potyviridae*, and *Secoviridae* (Figure 3). Members of *Hypoviridae*, *Potyviridae*, and *Secoviridae* are positive-sense, single-stranded RNA (+ssRNA) viruses [34,35,36]. The genomes of FgV1 and RnFV1 have been confirmed to be +ssRNA in nature [4,21]. Moreover, recent research has proposed to establish new family Fusarividae to accommodate FgV1 and RnFV1 [4]. Therefore, it is assumed that SsFV1 has a +ssRNA genome and tentatively placed in the newly proposed family Fusarividae.

Several +ssRNA viruses have been characterized in *S. sclerotiorum* and mainly grouped in existing families of *Narnaviridae* [10,17,18,19], *Hypoviridae* [20,21,22,23], *Endornaviridae* [24], and *Alphaflexiviridae* [16,23]. However, SsFV1 has markedly different features from previously reported +ssRNA viruses in *S. sclerotiorum*. SsFV1 comprises four ORFs and encodes four putative proteins, whereas all reported +ssRNA viruses of *S. sclerotiorum* contain only one ORF that encodes a putative polyprotein or RdRp protein. Thus SsFV1 relatively enriches viral resource in *S. sclerotiorum*.

Although SsFV1 is phylogenetically related to FgV1, RnFV1, and PrRV1, SsFV1 is distinct in three genome features compared to the other three viruses (Figure 2). First, SsFV1 has the largest genome with 7754 nts in length excluding the 3'-terminal poly(A) tail, whereas the genome lengths of FgV1, RnFV1, and PrRV1 are <7000 nts. In addition, the 418 nts long 3'-UTR of SsFV1 is longer than those of other members (3'-UTRs are <100 nts) in family Fusarividae. Second, the largest ORF (ORF1) encodes a polyprotein containing RdRp and helicase domains, a characteristic shared. However, SsFV1 and FgV1 have four putative ORFs, while RnFV1 and PrRV1 have only two ORFs. Third, the high A + U content (usually >60%) is a characteristic feature of mitovirus genomic RNA [37]. It is interesting that SsFV1 (61.3%) has higher A + U content and is richer in A+U content than FgV1 (48.5%), PrRV (50.2%), and RnFV1 (53.4%). 

### 3.4. SsFV1 Has No Clear Impact on S. sclerotiorum

**Figure 4:**
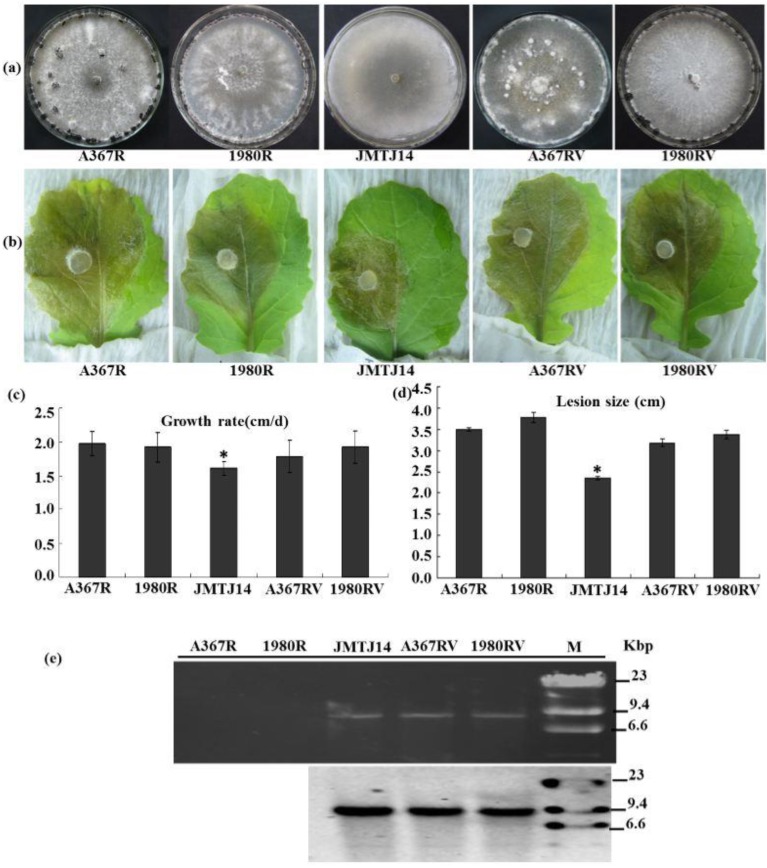
Biological properties of SsFV1-infected strains of *S. sclerotiorum*. (**a**) Colony morphology of *S. sclerotiorum* strains JMTJ14, A367R (EP-1PNA367R), 1980R, and two new representative SsFV1-infected strains A367RV (EP-1PNA367RV), 1980RV. All *S. sclerotiorum* strains were grown on PDA for 20 days at 20 °C prior to photography; (**b**) and (**d**) Virulence assays of *S. sclerotiorum* strains listed under detached leaves in (**b**); Assays were performed on detached rapeseed leaves. The diameters of disease lesion size were measured at 3 days post-inoculation (dpi); (**c**) Hyphal growth rates of *S. sclerotiorum* individual strains. Growth rates were examined on PDA at 20 °C; (**e**) Detection of SsFV1 in individual strains by dsRNA extraction and Northern blot analysis. dsRNA, the replicative form of SsFV1, was extracted and resolved by electrophoresis in a denaturing 1% agarose gel, transferred to a membrane, and probed with specific probe JRP680 (corresponding nt positions 2307–2986) for SsFV1 RNA. The band of about 7.8 kbp in size was clearly detected with dsRNA extraction and Northern blotting. Asterisks indicate a significant difference among strains of *S. sclerotiorum* according to the Student t test, *p* = 0.01.

Strain JMTJ14, which harbors a +ssRNA mycovirus SsFV1, is incapable of producing sclerotia on PDA and carrot media. Compared to virus-free strains, strain JMTJ14 is less virulent on its host. To determine whether mycovirus SsFV1 is involved in the abnormal phenotype of strain JMTJ14, mycovirus elimination and horizontal transmission tests were performed via conventional methods under laboratory conditions. SsFV1 was transmitted successfully from strain JMTJ14 to strains Ep-1PNA367R and 1980R via hyphal contact, but virus elimination experiments were not successful. This result was further confirmed by dsRNA extraction and Northern blot technique (Figure 4e). The newly SsFV1-infected strains Ep-1PNA367RV and 1980RV showed normal colony morphology and growth rate, which were similar to strain Ep-1PNA367R and 1980R (Figure 4a,c). Virulence tests suggested that SsFV1-infected strains Ep-1PNA367RV and 1980RV can cause larger lesions on detached rapeseed leaves than strain JMJT-14, but are not significantly different from their original strains Ep-1PNA367R and 1980R (Figure 4b,d). These results thus indicate that SsFV1 has no clear impact on *S. sclerotiorum*. The reason for sclerotial formation defect in strain JMTJ14 needs to be further exploited.

The biological roles of members of family Fusarividae are diverse in their hosts. Although the sequence of PrRV (accession number YP_009052456) recently became available from the NCBI virus database, its biological roles remain unknown. FgV1 is responsible for reducing mycotoxin production and virulence of *F. graminearum* strain DK21 [38], while RnFV1 infects the natural host asymptomatically [4]. Moreover, the host-virus interaction system of *Fusarium*-FgV1 has been studied, and some of the responses of *Fusarium* genes to virus infection were recently identified [39,40,41,42,43,44]. In the present study, although strain JMTJ14 is incapable of producing sclerotia in PDA or carrot medium, SsFV1 is apparently not responsible for the deficiency in sclerotial formation in strain JMTJ14 of *S. sclerotiorum*. Similar to virus-free strains, newly SsFV1-infected heterologous strains have are highly virulence on their host. Therefore, SsFV1 appears to be associated with latent infection in *S. sclerotiorum*. Deficiency in sclerotia production may be a property of strain JMTJ14 itself. In addition, SsFV1 can horizontally transfer from strain JMTJ14 to two vegetatively incompatible strains, Ep-1PNA367R and 1980R, which suggests that SsFV1 has the potential of breaking vegetative incompatibility barriers. 

We also attempted to eliminate the SsFV1 from the strain JMTJ14 via methods of protoplast regeneration, hyphal tipping isolation, and chemotherapy applications, but all attempts for virus elimination were unsuccessful and SsFV1 was stably retained in strain JMTJ14 of *S. sclerotiorum*.

## 4. Conclusions 

Various mycoviruses have been discovered and showed great diversity in populations of *S. sclerotiorum*. It is particularly important that some of hypovirulence-associated mycoviruses have great potential for virocontrol of stem rot of oilseed rape under natural condition [1,8]. In the present study, a newly described mycovirus, SsFV1, was isolated from virulent strain JMTJ14 of *S. sclerotiorum* and its full genome sequence (7754 nts in length) was determined. Our experimental data clearly point out that SsFV1 has a positive sense single-stranded RNA genome and is closely related to two previously reported mycoviruses, RnFV1 and FgV1, which belong to the proposed family Fusarividae. Horizontal transmission experiment reveals that SsFV1 is associated with latent infection of its host. This is the first report of a fusarivirus in *S. sclerotiorum* and our study will enhance the understanding of mycovirus diversity in filamentous fungi.

## Supplementary Files

Supplementary File 1

## References

[1] Xie J., Jiang D. (2014). New insights into mycoviruses and exploration for the biological control of crop fungal diseases. Annu. Rev. Phytopathol..

[2] Liu L., Xie J., Cheng J., Fu Y., Li G., Yi X., Jiang D. (2014). Fungal negative-stranded RNA virus that is related to bornaviruses and nyaviruses. Proc. Natl. Acad. Sci. USA.

[3] Ghabrial S.A., Suzuki N. (2009). Viruses of plant pathogenic fungi. Annu. Rev. Phytopathol..

[4] Zhang R., Liu S., Chiba S., Kondo H., Kanematsu S., Suzuki N. (2014). A novel single-stranded RNA virus isolated from a phytopathogenic filamentous fungus, *Rosellinia necatrix*, with similarity to hypo-like viruses. Front. Microbiol..

[5] Ghabrial S.A., Castón J.R., Jiang D., Nibert M.L., Suzuki N. (2015). 50-plus years of fungal viruses. Virology.

[6] Pearson M.N., Bailey A.M. (2013). Viruses of *Botrytis*. Adv. Virus Res..

[7] Liu H., Fu Y., Jiang D., Li G., Xie J., Peng Y., Yi X., Ghabrial S.A. (2009). A novel mycovirus that is related to the human pathogen Hepatitis E virus and *rubi-like* viruses. J. Virol..

[8] Bolton M.D., Thomma B.P.H.J., Nelson B.D. (2006). *Sclerotinia sclerotiorum* (Lib.) de Bary: Biology and molecular traits of a cosmopolitan pathogen. Mol. Plant. Pathol..

[9] Jiang D., Fu Y., Li G., Ghabrial S.A. (2013). Viruses of the plant pathogenic fungus *Sclerotinia sclerotiorum*. Adv. Virus Res..

[10] Xu Z., Wu S., Liu L., Cheng J., Fu Y., Jiang D., Xie J. (2015). A mitovirus related to plant mitochondrial gene confers hypovirulence on the phytopathogenic fungus *Sclerotinia sclerotiorum*. Virus Res..

[11] Yu X., Li B., Fu Y., Jiang D., Ghabrial S.A., Li G., Peng Y., Xie J., Cheng J., Huang J., Yi X. (2010). A geminivirus-related DNA mycovirus that confers hypovirulence to a plant pathogenic fungus. Proc. Natl. Acad. Sci. USA.

[12] Yu X., Li B., Fu Y., Xie J., Cheng J., Ghabrial S.A., Li G., Yi X., Jiang D. (2013). Extracellular transmission of a DNA mycovirus and its use as a natural fungicide. Proc. Natl. Acad. Sci. USA.

[13] Liu H., Fu Y., Jiang D., Li G., Xie J., Cheng J., Peng Y., Ghabrial S.A., Yi X. (2010). Widespread horizontal gene transfer from double-stranded RNA viruses to eukaryotic nuclear genomes. J. Virol..

[14] Liu H., Fu Y., Xie J., Cheng J., Ghabrial S.A., Li G., Peng Y., Yi X., Jiang D. (2012). Evolutionary genomics of mycovirus-related dsRNA viruses reveals cross-family horizontal gene transfer and evolution of diverse viral lineages. BMC Evol. Biol..

[15] Xiao X., Cheng J., Tang J., Fu Y., Jiang D., Baker T.S., Ghabrial S.A., Xie J. (2014). A novel partitivirus that confers hypovirulence on plant pathogenic fungi. J. Virol..

[16] Xie J., Wei D., Jiang D., Fu Y., Li G., Ghabrial S., Peng Y. (2006). Characterization of debilitation-associated mycovirus infecting the plant-pathogenic fungus *Sclerotinia sclerotiorum*. J. Gen. Virol..

[17] Xie J., Ghabrial S.A. (2012). Molecular characterization of two mitoviruses co-infecting a hypovirulent isolate of the plant pathogenic fungus *Sclerotinia sclerotiorum*. Virology.

[18] Khalifa M.E., Pearson M.N. (2013). Molecular characterization of three mitoviruses co-infecting a hypovirulent isolate of *Sclerotinia sclerotiorum* fungus. Virology.

[19] Khalifa M.E., Pearson M.N. (2014). Molecular characterisation of novel mitoviruses associated with *Sclerotinia sclerotiorum*. Arch. Virol..

[20] Xie J., Xiao X., Fu Y., Liu H., Cheng J., Ghabrial S.A., Li G., Jiang D. (2011). A novel mycovirus closely related to hypoviruses that infects the plant pathogenic fungus *Sclerotinia sclerotiorum*. Virology.

[21] Marzano S.Y., Hobbs H.A., Nelson B.D., Hartman G.L., Eastburn D.M., McCoppin N.K., Domier L.L. (2015). Transfection of *Sclerotinia sclerotiorum* with *in vitro* transcripts of a naturally occurring interspecific recombinant of *Sclerotinia sclerotiorum* hypovirus 2 significantly reduces virulence of the fungus. J. Virol..

[22] Khalifa M.E., Pearson M.N. (2014). Characterisation of a novel hypovirus from *Sclerotinia sclerotiorum* potentially representing a new genus within the *Hypoviridae*. Virology.

[23] Hu Z., Wu S., Cheng J., Fu Y., Jiang D., Xie J. (2014). Molecular characterization of two positive-strand RNA viruses co-infecting a hypovirulent strain of *Sclerotinia sclerotiorum*. Virology.

[24] Khalifa M.E., Pearson M.N. (2014). Molecular characterisation of an endornavirus infecting the phytopathogen *Sclerotinia sclerotiorum*. Virus Res..

[25] Schafer M.R., Kohn L.M. (2006). An optimized method for mycelial compatibility testing in *Sclerotinia sclerotiorum*. Mycologia.

[26] BLAST program software. http://blast.ncbi.nlm.nih.gov/Blast.cgi.

[27] Potgieter A.C., Page N.A., Liebenberg J., Wright I.M., Landt O., van Dijk A.A. (2009). Improved strategies for sequence-independent amplification and sequencing of viral double-stranded RNA genomes. J. Gen. Virol..

[28] GenBank database. http://www.ncbi.nlm.nih.gov/genomes.

[29] Transfac software. http://www.genome.jp/tools/motif/.

[30] InterProscan software. http://www.ebi.ac.uk/Tools/pfa/iprscan.

[31] Multiple sequence alignment MAFFT software. http://mafft.cbrc.jp/alignment/server/.

[32] Tamura K., Stecher G., Peterson D., Filipski A., Kumar S. (2013). MEGA6: Molecular evolutionary genetics analysis version 6.0. Mol. Biol. Evol..

[33] Zhang L., Fu Y., Xie J., Jiang D., Li G., Yi X. (2009). A novel virus that infecting hypovirulent strain XG36–1 of plant fungal pathogen *Sclerotinia sclerotiorum*. Virol. J..

[34] Nuss D.L., Hillman B.I., King A.M.Q., Elliot L., Adams M.J., Carstens E.B. (2011). Family *Hypoviridae*. Virus taxonomy. Ninth Report of the International Committee on Taxonomy of Viruses.

[35] Adams M.J., Zerbini F.M., French R., Rabenstein F., Stenger D.C., Valkonen J.P.T., King A.M.Q., Elliot L., Adams M.J., Carstens E.B. (2011). Family Potyviridae. Virus Taxonomy. Ninth Report of the International Committee on Taxonomy of Viruses.

[36] Sanfaçon H., Iwanami T., Karasev A.V., van der Vlugt R., Wellink J., Wetzel T., Yoshikawa N., King A.M.Q., Elliot L., Adams M.J., Carstens E.B. (2011). Family *Secoviridae*. Virus taxonomy. Ninth Report of the International Committee on Taxonomy of Viruses.

[37] Hillman B.I., Cai G. (2013). The family *narnaviridae*: simplest of RNA viruses. Adv. Virus Res..

[38] Kwon S.J., Lim W.S., Park S.H., Park M.R., Kim K.H. (2007). Molecular characterization of a dsRNA mycovirus, Fusarium graminearum virus-DK21, which is phylogenetically related to hypoviruses but has a genome organization and gene expression strategy resembling those of plant potex-like viruses. Mol. Cells.

[39] Lee K.M., Yu J., Son M., Lee Y.W., Kim K.H. (2011). Transmission of *Fusarium boothii* mycovirus via protoplast fusion causes hypovirulence in other phytopathogenic fungi. PLoS ONE.

[40] Lee K.M., Cho W.K., Yu J., Son M., Choi H., Min K., Lee Y.W., Kim K.H. (2014). A comparison of transcriptional patterns and mycological phenotypes following infection of *Fusarium graminearum* by four mycoviruses. PLoS ONE.

[41] Son M., Lee K.M., Yu J., Kang M., Park J.M., Kwon S.J., Kim K.H. (2013). The *HEX1* gene of *Fusarium graminearum* is required for fungal asexual reproduction and pathogenesis and for efficient viral RNA accumulation of Fusarium graminearum virus 1. J. Virol..

[42] Cho W.K., Yu J., Lee K.M., Son M., Min K., Lee Y.W., Kim K.H. (2012). Genome-wide expression profiling shows transcriptional reprogramming in *Fusarium graminearum* by Fusarium graminearum virus 1-DK21 infection. BMC Genomics.

[43] Yu J., Lee K.M., Son M., Kim K.H. (2015). Effects of the deletion and over-expression of *Fusarium graminearum* gene *FgHal2* on host response to mycovirus Fusarium graminearum virus 1. Mol. Plant Pathol..

[44] Cho W.K., Lee K.M., Yu J., Son M., Kim K.H. (2013). Insight into mycoviruses infecting *Fusarium* species. Adv. Virus Res..

