# Supplementary material for: Molecular Characterization of a Novel Positive-Sense, Single-Stranded RNA Mycovirus Infecting the Plant Pathogenic Fungus Sclerotinia sclerotiorum
Source: Viruses. 2015 May 21;7(5):2470–84. doi: 10.3390/v7052470 (PMC4452915; doi:10.3390/v7052470)
Supplement: Supplementary File 1 [file viruses-07-02470-s001.pdf]

Supplementary Materials

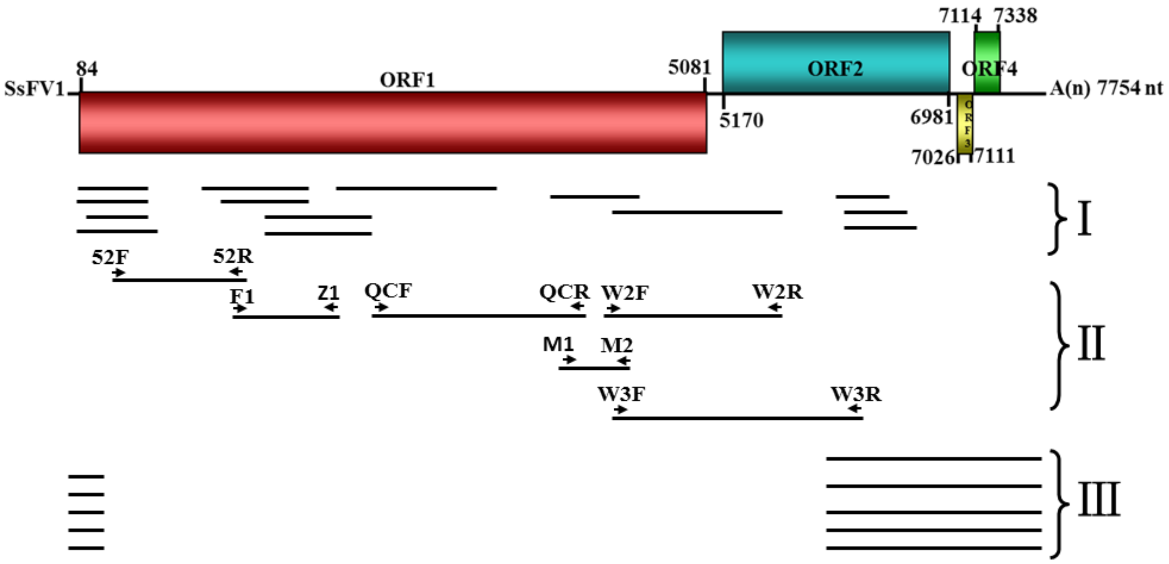

Figure S1. Schematic representation of the strategy used for SsFV1 full cDNA sequence. Partial cDNA clones (I) were obtained using tagged random dN6 primers and denatured dsRNA as a template. dsRNA-specific primers (Table S1) were designed based on the sequences of the initial cDNA clones generated by random primers and used in RT-PCR amplification to produce cDNA clones (II) for dsRNA regions that was not covered by the initial cDNA clones. The 5' and 3' terminal clones (III) were synthesized using the RACE method described by Xie *et al.* (2006).

Table S1. Oligonucleotide primers/adaptor used in this study.

Name	Sequence (5' → 3')	Position in Genome of SsFV1
To cDNA clone		
52R	AAATTTGTACCATCTTGTAGCC	356–377
52F	AAATTTGTACCATCTTGTAGCCAGC	1417–1441
F1	ATCAATGAAGATGGCAAGACAATAA	1374–1398
Z1	TAGAGCCCCTTCCCTTTTTCAT	2427–2448
QCF	ATCATTATGCTGGAGATTGTAC	2132–2153
QCR	CTTTGGTAACTCAATTTCCGTTG	4022–4044
M1	CTTATTTGTGCTGGATGAATCTC	3928–3950
M2	CACCGTTATTAGTGCGACCAGT	4454–4475
W2F	ACGCCTAATTTTACCAGAAATG	4399–4421
W2R	AGGAGCTGCGGCCGCTGCA	5461–5482
W3F	GAAAAGAATTTGACAACTGAACA	4664–4686
W3R	GGTACAGTTTTGGTTTCCTG	6221–6242
tagged random primers-dN6	CGATCGATCATGATGCAATGCNNNNNN	
Complementary primer for dN6	CGATCGATCATGATGCAATGC	
To detect cDNA inserts in the pMD18-T vector		
M13F-47	CGCCAGGGTTTTCCCAGTCACGAC	pMD18-T
M13R-48	AGCGGATAACAATTTCACACAGGA	pMD18-T

Table S2. The information of viruses or genes used in the present study.

Virus Name	Acronym	RdRp Identities (%)	Helicase Identities (%)	Accession Number	Family
Sclerotinia sclerotiorum fusarivirus 1	SsFV1	-	-	KP842791	
Fusarium graminearum dsRNA mycovirus 1	FgV1	133/266 (50%)	95/291 (33%)	AAT07067	
Penicillium roqueforti dsRNA mycovirus 1	PrRV1	128/266 (48%)	110/320 (34%)	AII99895	Fusariviridae
Rosellinia necatrix fusarivirus 1	RnFV1	118/259 (46%)	101/294 (34%)	YP_009047147	
Grapevine associated mycovirus-2	GaMV2	124/255(49%)	-	ADO60939	
Cryphonectria hypovirus 1	CHV1	63/266 (24%)	41/164 (25%)	NP_041091	
Cryphonectria hypovirus 2	CHV2	43/199 (22%)	72/295 (24%)	NP_613266	
Cryphonectria hypovirus 3	CHV3	69/199 (35%)	77/310 (25%)	NP_051710	
Cryphonectria hypovirus 4	CHV4	84/244 (34%)	79/303 (26%)	YP_138519	
Sclerotinia sclerotiorum hypovirus 1	SsHV1	76/244 (31%)	90/362 (25%)	AEL99352	*Hypoviridae*
Sclerotinia sclerotiorum hypovirus 2	SsHV2	87/251 (35%)	19/71 (27%)	AIA61616	
Fusarium graminearum hypovirus 1	FgHV1	69/234 (29%)	-	YP_009011065	
Valsa ceratosperma hypovirus 1	VcHV1	81/244 (33%)	-	BAM08994	
Phomopsis longicolla hypovirus 1	PlHV1	77/244 (32%)	-	AIG94930	
Potato virus Y	PVY	49/209 (23%)	64/293 (22%)	NP_056759	
Potato virus V	PVV	48/195 (25%)	69/320 (22%)	NP_659008	
Zantedeschia mild mosaic virus	ZmMV	58/209 (28%)	-	YP_002321507	
Daphne mosaic virus	DMV	52/192 (27%)	31/149 (21%)	YP_610949	
Potato virus A	PVA	54/211 (26%)	-	NP_659729	*Potyviridae*
Barley mild mosaic virus	BmMV	47/135 (35%)	62/298 (21%)	NP_734299	
Wheat yellow mosaic virus	WyMV	43/126 (34%)	-	CAB59641	
Oat mosaic virus	OMV	41/126 (33%)	26/131 (20%)	NP_734281	
Maize chlorotic dwarf virus	McDV	48/208 (23%)	-	NP_734456	
Apple latent spherical virus	ALSV	49/190 (26%)	-	NP_734022	
Cherry rasp leaf virus	CLV	47/190 (25%)	-	YP_081454	
Satsuma dwarf virus	SDV	59/218 (27%)	-	NP_734025	*Secoviridae*
Chocolate lily virus A	CLVA	57/225 (25%)	-	YP_004940021	
Strawberry mottle virus	SMV	42/185 (23%)	19/82 (23%)	NP_733954	
Black raspberry necrosis virus	BRNV	40/181 (22%)	15/55 (27%)	YP_654561	
Vibrio vulnificus	-	-	71/317 (22%)	NP_761612	Bacterium
Pseudoalteromonas sp.	-	-	76/338 (22%)	WP_008133989	Bacterium
Vibrio owensii	-	-	79/322 (25%)	WP_039842595	Bacterium
Schizosaccharomyces cryophilus	-	-	78/326 (24%)	EPY51743	Yeast
Aliivibrio fischeri	-	-	86/332 (26%)	WP_005419796	Bacterium
Saprolegnia diclina VS20	-	-	71/338 (21%)	XP_008605027	Saprolegniaceae
Photobacterium angustum	-	-	73/316 (23%)	WP_039872432	Bacterium

"-" means no identities.
